# Carotenoids dispersed in gypsum rock as a result of algae adaptation to the extreme conditions of the Atacama Desert

**DOI:** 10.1038/s41598-024-75526-7

**Published:** 2024-10-13

**Authors:** P. Vítek, C. Ascaso, O. Artieda, J. Wierzchos

**Affiliations:** 1 Global Change Research Institute of the Czech Academy of Sciences, Bělidla 986/4a, Brno, 603 00 Czech Republic; 2 Museo Nacional de Ciencias Naturales, CSIC, c/ Serrano 115 dpdo, Madrid, 28006 Spain; 3 Departamento Biología Vegetal, Ecología y Ciencias de la Tierra, and IACYS, Universidad de Extremadura, Plasencia, 10600 Spain

**Keywords:** Photopigments, Extremophiles, Astrobiology, Geomicrobiology, Raman imaging, Biomarkers, Biophysics, Plant sciences, Biogeochemistry, Ecology

## Abstract

The high-altitude pre-Andean region of the Atacama Desert is characterized by its stark volcanic rock formations and unique hydrothermal gypsum outcrops (gypcrete) that it hosts. This study delves into the biomolecular composition of the endolithic phototrophic microbes that thrive within these gypcretes. Using advanced Raman spectroscopy techniques, including Raman imaging (complemented by microscopic and 3D microscopic observations), herein we unveil new insights into the adaptive strategies of these gypsum-inhabiting algae. Our Raman imaging results provide a detailed chemical map of carotenoids associated with microbial colonization. This map reveals a significant gradient in pigment content, highlighting a critical survival mechanism for algae and cyanobacteria in this polyextreme environment. Intriguingly, we detected signals for carotenoids not only in the algae-colonized layer, but also deeper within the gypsum matrix - indicating pigment migration following cell disruption. In addition, we conducted an in-depth analysis of individual algal cells from the Trebouxiaceae family, noting their color variations from green to orange, plus describing the spectral differences in detail. This investigation identified in-vivo pigments (carotenoids, chlorophyll) and lipids at the cellular level, offering a comprehensive view of the molecular adaptations enabling life in one of the Earth’s most extreme habitats.

## Introduction

The Preandean zone of the Atacama Desert occurs in the northeastern area of the desert at altitudes higher that the core parts (i.e., ~ 3000 m a.s.l.). As a result, the area is among one of the Earth’s most intensively sun-irradiated locations^1^. It includes the high level of UV radiation, which is reported for all cloudless and high-altitude Andean regions; with very low relative humidity (RH) values, and a low-ozone column (UV index 43.3, see Cabrol et al.^2^). The yearly precipitation at the studied site has been reported to be 27.1 mm^3^, while the mean annual RH recorded by Wierzchos et al.^4^ was 16.5%; this being one of the lowest measured values within the Atacama Desert^5^. Together with the high evapotranspiration rate of 2920 mm year^−1^ recorded in the nearby Salar de Atacama basin, the region can be characterized as hyperarid; with an aridity index (AI) of 0.009, thus creating polyextreme abiotic stresses upon the biota with which they have to cope.

Raman spectroscopy is known to be a powerful tool for nondestructive in-vivo characterization of biomolecules associated with extremophilic phototrophs, with emphasis on pigment analysis^6–10^. The technique has been applied for the analysis of gypsum inhabiting endolithic communities from the Atacama Desert^11^, including Raman imaging applied to those algae from gypcrete in the Cordon de Lila area^4,12^. The Authors describe the gradient of the carotenoid Raman signal intensity distribution, with its enhancement close to the surface, which was interpreted as an adaptation mechanism to excessive solar irradiation. Here, while studying the same geomicrobiological system, we focused on the detailed in-vivo spectral characterization of algal cells differing visually in their color; as well as on the imaging of the carotenoids, including zones outside of the colonization zone. Cyanobacteria may occur under algae in some positions within the cryptoendolithic habitat^4^, but we did not focused on cyanobacteria in this study.

Microalgae are known to contain lipid storage bodies, which can form a significant portion of the algal mass, and which can be detected by Raman spectroscopy^13^. From a biotechnological point of view, algal lipids are of special importance^14^. They are considered an important source of biofuels, which also do not compete for land area with its other demands, i.e., food production. Using Raman spectroscopy, various algal strains were examined in order to obtain information about their lipid composition, e.g., *Botryococcus sudeticus*, *Chlamydomonas* sp., *Trachydiscus minutus*^13^. Aside from the conventional Raman spectroscopy, application of coherent anti-stokes Raman scattering microscopy may be favored for imaging of lipids and was employed for the algal lipid bodies by Cavonius et al.^15^. In this study, both pigment and lipid composition of algae were monitored using Raman spectroscopy in vivo in their native microhabitat.

## Results

### Raman spectroscopy – pigments

**Fig. 1:**
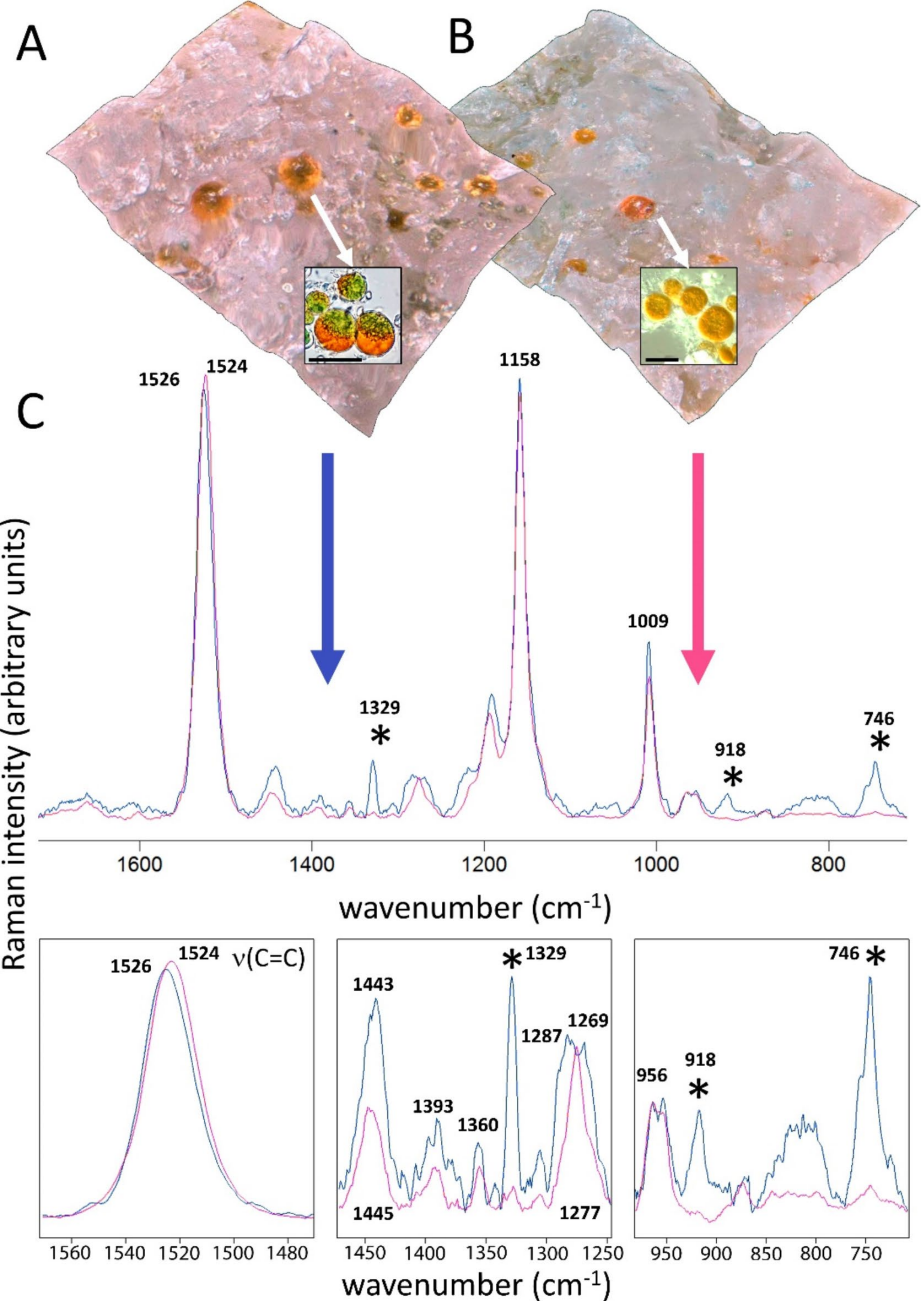
3D microscopic images and Raman spectra from the algae: (**A**) an image of orange-green algal cells embedded in gypsum, (B) an image of orange algal cells in gypsum, (**B**) Raman spectra obtained at the two types of cells (regions of interest magnified below), obviously differing in chlorophyll Raman features (major features indicated by *) and slight shift in carotenoid ν(C=C) band. Note that the blue spectrum corresponds to orange-green cells in A), while the magenta spectrum corresponds to orange cells in B). Scale bars = 20 μm. Laser wavelength 785 nm.

**Fig. 2:**
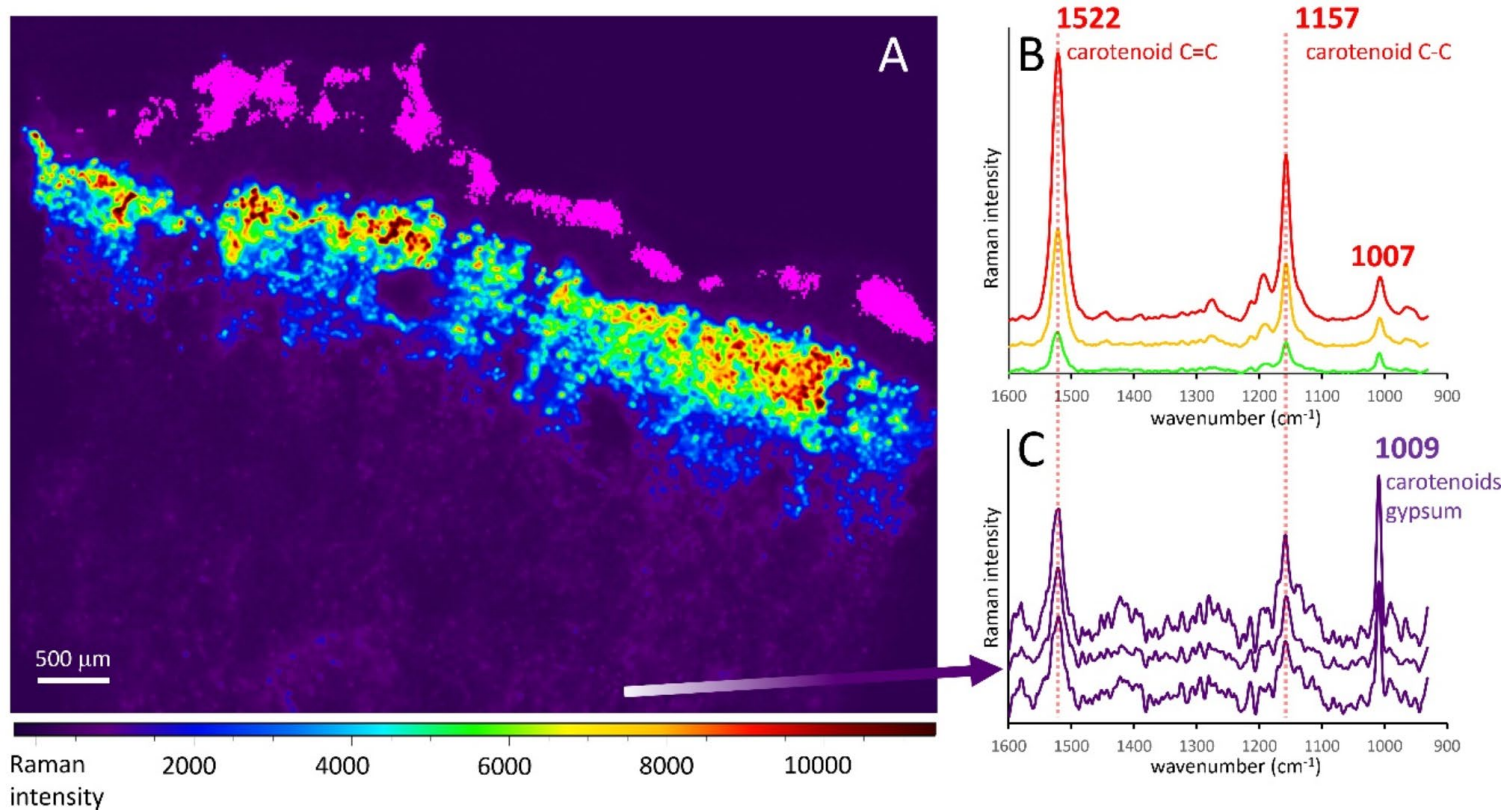
Raman imaging of the cryptoendolithic colonization in crosscut of gypcrete. (**A**) The rainbow scale corresponds to the intensity of carotenoid ν(C=C) band. The averaged spectra extracted from the zones of various intensity are presented: the colonized zone in (**B**), and gypcrete matrix with carotenoid signal in (**C**). Pixels in pink color at the upper part were deleted and represent Raman signal from the resin used for the sample preparation, that caused an oversaturation of the detector. Laser wavelength 514.5 nm.

Typical Raman spectra obtained on the algal cells are presented in Fig. 1. Two types of cells are compared – orange-green (Fig. 1A) and red-orange without visible green pigmentation (Fig. 1B). The employed red laser line (785 nm) allows for detection of wider variety of pigments and other chemicals compared to green lasers, that are excellent for resonance Raman enhancement of carotenoids (see the next chapter). The Raman spectroscopy shows differences in pigment composition (Figs. 1C and 2). Carotenoid Raman features dominate both of the spectra with the in-phase ν_1_(C=C) and ν_2_(C–C) stretching vibrations of the polyene chain located at 1523–1526 cm^−1^ and 1158–1159 cm^−1^, respectively. The feature of medium intensity occurs around 1009 cm^−1^, corresponding to the in-plane rocking modes of the CH_3_ groups attached to the polyene chain^16,17^. The small shifts of the two strong Raman bands within the wavenumber range, mentioned above, were observed between the two types of cells; with the higher values detected in the orange-green cells. Simultaneously, clear corroborative chlorophyll features were detected in these cells, with major bands located at 746, 918, and 1329 cm^−1^.

Significantly higher intensity of Raman carotenoid spectral features were detected within the red-orange cells in the uppermost zone of colonization, with substantially lower chlorophyll intensity compared to the green or orange-green cells from the lower parts. This is evident from the Raman spectra shown in Fig. 1C. The Raman intensity gradient of carotenoids was confirmed by Raman imaging of the whole thin section from the cross-cut gypcrete sample (Fig. 2).

## Raman spectroscopy - carotenoid distribution within gypcrete

The carotenoid signal, obtained by resonance Raman imaging using green laser line (514.5 nm), produced specific zonation within the gypcrete crosscut (Fig. 2). In the colonization zone, ~ 1–2 mm beneath the surface, we see the gradient of ν_1_(C=C) band intensity, with the most intensive Raman signal occurring at the uppermost parts of the colony (umbrella-like pattern). Interestingly, we also see a signal of lower intensity, yet clearly detectable, attributed to carotenoids several mm beneath the visible colonization by algal cells. The signal clearly comes from carotenoids dispersed in the gypsum matrix. The different intensity ratio of the ν_1_(C=C) and ν_2_(C-C) bands, respectively, that can be observed between the spectra in Figs. 1 and 2 is caused by the two different excitation wavelengths.

## Raman Spectroscopy - lipids

**Fig. 3:**
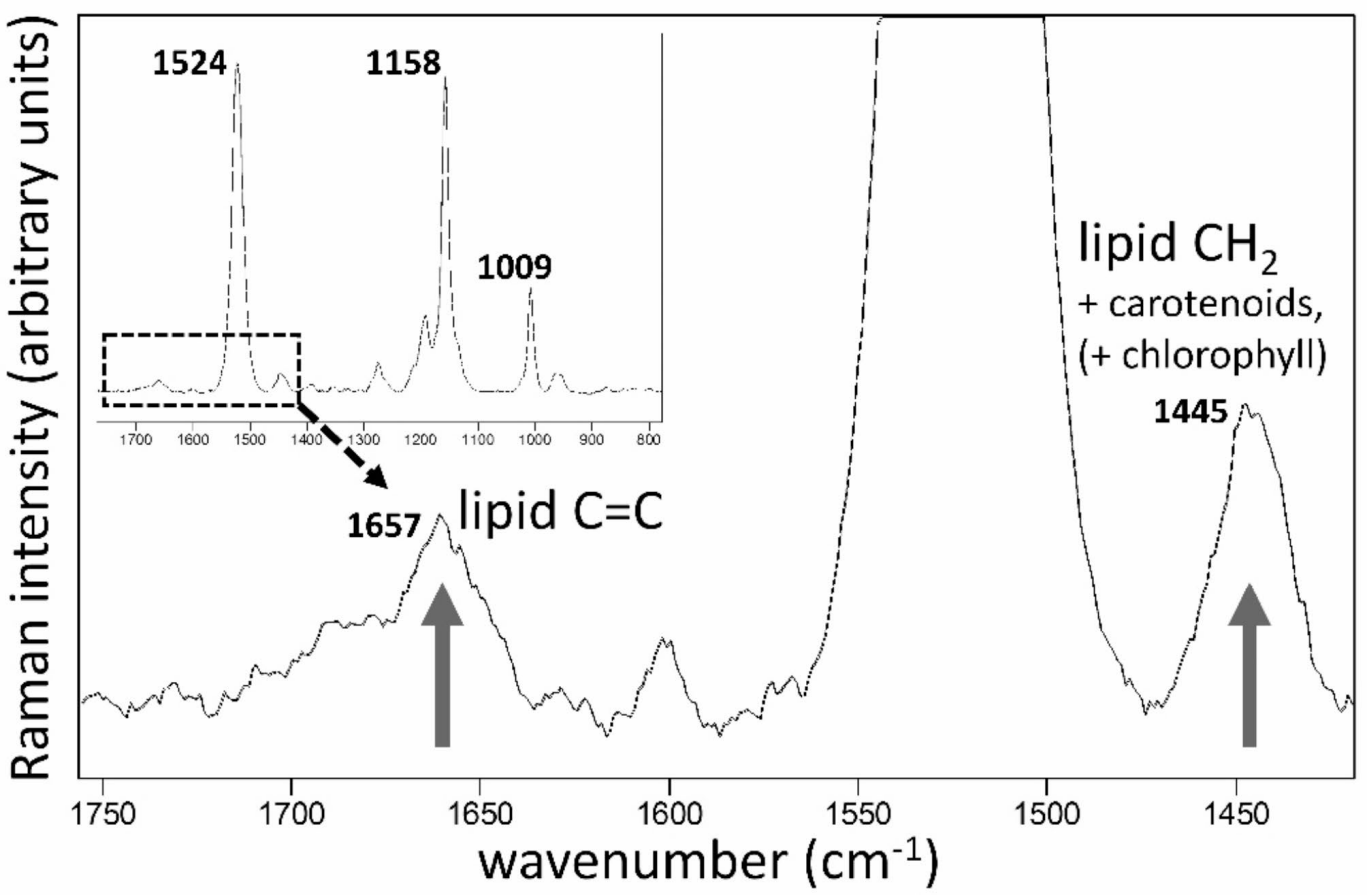
Raman spectroscopic features of lipids at 1657 cm^-1^ due to C=C stretching, and 1445 cm^-1^ assigned to CH_2_ scissoring in lipids. The later is at a similar position as the weak Raman features of carotenoids and chlorophyll. Laser wavelength 785 nm.

Raman spectra of lipids are characterized by a couple of corroborative Raman bands in the 1000–1800 cm^−1^ range^18^. The band located around 1657 cm^−1^ is assigned to C=C stretching vibrational mode. The band at 1445 cm^−1^ is mainly due to scissoring of CH_2_ in lipids. Both bands, assigned to lipids, were detected as weak features in the spectral record obtained from algae within the gypsum dominated by strong carotenoid features (Fig. 3). The ratio of these two bands can then be used as an indicator of lipid unsaturation^13,19,20^. The contribution of the carotenoid Raman signal to the band located at 1445 cm^−1^ also has to be taken into account. Within the β-carotene standard, the intensity of the band at 1445 cm^−1^ is 1/33 of the intensity of the ν(C=C) band. After correction of the carotenoid contribution of the band around 1445 cm^−1^, an intensity ratio of the unsaturated/saturated band (I_1657_/I_1445_) was 1.26 (according to Samek et al., 2011^19^, it corresponds to the iodine value IV ≈ 150).

## Discussion

An umbrella-like pattern of carotenoid distribution, as detected by Raman imaging (Fig. 2), was also presented in the earlier studies of the same material (see^4,12,21^. Hence, it was observed repeatedly from the different sampled zones of the same material. It has been interpreted as a biomolecular adaptation of the algae to excessive solar radiation, which occurs in the pre-andean region of the Atacama Desert^1^. We hypothesize that lower positioned cells benefit from the shielding provided by the cells in higher positions. On the other hand, we do not expect that the algal cells located in the uppermost parts might benefit from the photosynthetic activity of cells located below, which have both denser chloroplasts throughout the cytoplasm^4^, and a higher chlorophyll content, as described herein. Zonation of photopigments and phototrophic microorganisms is described from the wet gypsum environment of solar salterns^22^. Though very different environment and composition of microbiota, there are two aspects that may be analogous in this study: 1) the enhanced carotenoid content relative to chlorophyll *a* within the uppermost layer, 2) the sequence of colors from orange/brown (top) to green located just beneath.

Employment of Raman spectroscopy for the assessment of algal lipids has increased, benefiting from its nondestructive, label-free nature, and the possibility to examine single cells^13,19,20^. Nevertheless, those studies are focused on the cultivated cultures of algae, rich in lipids. Here, in contrast, in vivo chemical detection of lipids within these native algal cells in their rock habitat has been achieved. Their preliminary detection was mentioned in Vítek and Wierzchos^23^. As far as we know, it is the first Raman spectroscopic demonstration of lipids in native endolithic community. We explain this observation as a consequence of very high lipid content in these algae cells that can be detected beside the biomolecular signal of much better Raman scatterers such as carotenoids.

Lipids can be produced by algae and stored in the form of subcellular structures called lipid bodies, lipid droplets, oil bodies, oil globules or oleosomes^24,25^. The terms are dependent on a particular community, and reflect the same structure. In this study we use the term lipid body. The major role of lipid bodies is either in lipid storage prior to the dormancy period, or when subjected to stressful environmental conditions^26,27^. Triacylglycerols, that form the basis of the lipid bodies, contain twice more energy compared to starch or protein per unit weight, hence represent an effective carbon and energy storage in eukaryotic cells, in general^27,28^. It was reported by Li et al.^29^, that *Chlamydomonas* mutant *pgd*1, containing a lesser amount of storage lipids readily lost its viability under nitrogen-deprived conditions. Another study reported about nitrogen starvation-induced formation of lipid bodies and conversion of membrane lipid acyl groups to triacylglycerol in green algae *Chlorella* sp^30^. Nevertheless, there are a variety of functions beyond energy storage^28^. It was reported, that triacylglycerides in the lipid bodies can serve as a temporary depot for acyl chains. These are removed from membrane structures under adverse conditions like osmotic stress, freezing, heat stress etc. in order to compensate changes in bilayer structures^28,31^. When environmental conditions change again, the acyl chains are released from the lipid bodies and are again used for membrane lipids synthesis and assembly. In this way, the cell bypasses the need for *de novo* fatty acid synthesis^28^. It could require more energy consumption, which in the extreme environmental conditions such as the studied zone of the Atacama Desert may be a crucial factor. Another role in cellular physiology are hypothesized based on the proteomics of lipid bodies^32,33^. These are cell signaling, protection, molecular transport etc^28^. It has also been demonstrated, that the algal lipid content can be stimulated by manipulating the solar light spectrum with color paints dissolved in water^34,35^.

Carotenoids are pigments involved in photosynthesis, photoprotection, and membrane stabilization. They are characterized by a system of long conjugated C=C bonds composed of isoprenoid units. One important role of xanthophyll carotenoids in very high PAR (photosynthetically active radiation) conditions is the dissipation of excess excitation energy in the xanthophyll cycle, which is considered a key photoprotective mechanism in higher plants and algae^36,37^. Thus, it prevents the formation of singlet oxygen (^1^O_2_), by rapidly quenching chlorophyll triplet states. Moreover, there is an important role for carotenoids to rapidly and directly scavenge singlet oxygen, if any is formed^38–41^. The radical-scavenging ability of carotenoids has been reported to be more effective with the higher conjugation of the polyene structure^42^.

Here, all the observed carotenoid band positions may be attributed to C-40 carotenoids, with ν_1_(C=C) band position 1522 cm^−1^ or higher, that can be attributed to xanthophyll carotenoids. Spectral differences observed between the spectra in Figs. 1 and 2, respectively result from the two different laser wavelengths used for excitation. The small shift of the ν_1_(C=C) wavenumber position was observed, with 1522 cm^−1^ detected when using 514.5 nm laser line and 1523–1526 cm^−1^ while the red (785 nm) laser was employed. We interpret this as a result of selective resonance Raman enhancement caused by the green excitation at 514.5 nm. Using this wavelength (514.5 nm) for excitation also caused the relative enhancement of the signal due to the ν_1_(C=C) bond vibration, leading in different intensity ratio of the two strongest bands when compared to the spectra obtained by 785 nm laser line.

We hypothesize that to defend themselves from excess radiation, algae at mature state within the upper parts of the colonized layer almost lose their green chloroplasts, as such, and become cells full of lipids that accumulate carotenoids, which results in an intensive orange color due to the high amount of them. In the end, these algae in a mature state come to be degraded, end up exploding and release lipids and dissolved carotenoids. As a result of this process, carotenoids, dissolved in lipids migrate within the substrate and are dispersed in the deeper parts in the mineral (gypcrete) environment.

## Conclusions

- A novel insight into the adaptation strategy of endolithic, gypsum-inhabiting algae from the polyextreme environment of the Atacama Desert is unveiled, providing fresh insights into their survival mechanisms.
- Carotenoids were discovered beneath the microbially colonized zone within the gypsum matrix, suggesting migration of these pigments following cell disruption.
- Comprehensive Raman spectroscopic analysis revealed distinct differences in carotenoid and chlorophyll composition between green-orange and orange algal cells of the Trebouxiaceae family, cohabiting the gypcrete rock.
- For the first time, in vivo Raman spectra of lipids within these algal cells are presented. We suggest lipids play an important role in release and migration of carotenoids within the gypsum substrate after cell death and disruption.

### Methods

#### Study site and samples

**Fig. 4:**
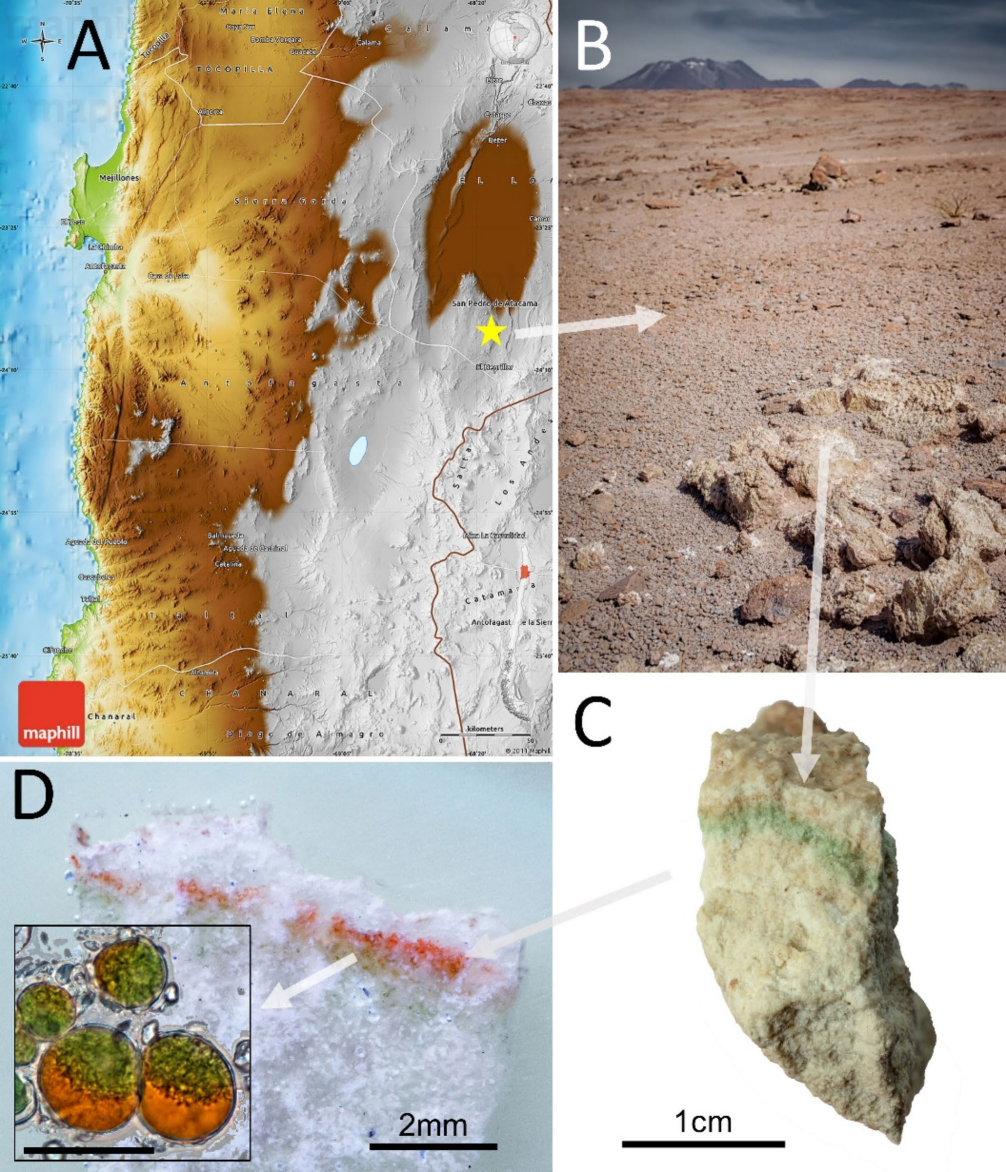
Sampling site close to the southern edge of Salar de Atacama (yellow star on the map (A) is formed by volcanic rocks with zones of gypcrete outcrops (B). Green-to-red layers of endolithic colonization can be found 1–5 mm below the surface (C). In (D) a slide cut from the gypcrete with intensively colored colonization and high magnification image with algal cells is depicted. Scale bar in the magnified image in D = 20 μm. The map in (A) was generated by Maphill under CC BY-ND license, www.maphill.com.

The sampling zone (23° 53′ S, 068° 08′ W; 2720 m a.s.l.) was located within the southern edge of the Salar de Atacama basin (Fig. 4A), in the north–south-trending depression of the Cordon de Lila range, in northern Chile. This depression is mostly covered by volcanic material, but large gypsum outcrops can be found in several locations (Fig. 4B). In the field, gypsum deposits were assessed for endolithic colonization by visual inspection for microbial pigments present in fractured samples (Fig. 4C). These pigments indicate the presence of cryptoendolithic (occupying pore spaces beneath the rock surface) and hypoendolithic (colonizing the undermost layer of the rock, *sensu* Wierzchos et al.^4,43^. microbial colonization, forming horizons beneath the surface and close to the bottom of the gypsum deposits. The gypsum rock fragments were collected in hyperarid conditions with air relative humidity (RH) of 10% and an air temperature (T) of 28 °C. They were stored in dry and dark conditions at room temperature until analysis. About 100 μm thick saw-cut cross-section was prepared for Raman imaging analysis (Fig. 4D).

Gypsum formations at the studied site are called gypcrete (according to the nomenclature of Horta^44^), and occur as hard layer deposits on the soil surface, interbedded between layers of ignimbrites and sometimes filling cracks in these rocks. Abundant colonization can be recognized visually in the field by green-to-orange pigmentation after cracking open of the gypcretes. The cryptoendolithic habitat appears beneath the 0.5–5 mm thin surface layer. The typical zonation was revealed to be due to orange (higher position) and green (lower position) algae, with cyanobacteria appearing together within the green layer. Moreover, cyanobacteria were found to occupy a hypoendolithic habitat, e.g., at the bottom position, close to the contact with the soil. For a detailed characterization of the gypsum formations and the microbial colonization by chlorophototrophic biota, see the comprehensive study by Wierzchos et al.^4^.

## Optical microscopy and 3D surface microscopy

A Zeiss AxioImager D1 microscope (Carl Zeiss, Oberkochen, Germany) equipped with a Plan-Apo 609/1.4 Zeiss oil-immersion objective was employed to obtain optical images of the algal cells.

In addition, we have used a Keyence VHX − 900 F (Keyence, UK) digital microscope with 100x magnification objective to scan the 3D surface structure of the algal cells embedded in the gypsum samples.

### Analyses of single microalgal cells by Raman spectroscopy

Microalgae cells, colonizing the cryptoendolithic habitat of gypcrete, were examined using point Raman analysis on an *InVia* spectrometer (Renishaw, Wotton-under-Edge, UK) equipped with a Leica confocal microscope. A 785 nm laser line was employed as a universal excitation wavelength capable of detecting a variety of pigments and other biomolecules, including lipids. The instrument was calibrated to a silicon Raman band at 521 cm^−1^. The point analysis was undertaken employing 50x magnification objective, and a 2 –5 s exposure time was set and accumulated 10 times. Laser power between 15 and 30 mW at the source was used.

The analyses were performed on transects of the gypcrete substrate stored in dark conditions at 20 °C until analysis. The Raman spectroscopy technique was chosen for its ability to analyse small amounts of biological material in situ.

### Raman imaging and Raman data processing

The same *InVia* spectrometer (Renishaw, Wotton-under-Edge, UK) equipped with a Leica confocal microscope was used in point-to-point scanning mode for the Raman imaging. The instrument was calibrated to a silicon Raman band at 521 cm^-1^. For imaging, an Ar laser line at 514.5 nm, with 10 mW power at the source, and a 1 s exposure time accumulated 1x time was employed at each point. Benefiting from the resonance Raman effect, a strong signal of carotenoids was obtained within the Raman imaging using a relatively short exposure time. As a result, a relatively large area was scanned at a high spatial resolution. The laser was focused using a 5x magnification Leica objective (NA = 0.12). Single spectra (averaged from 7 neighbor spectra) were extracted from the zones of interest to show the spectral differences. The Raman imaging data were acquired using Wire 3.4 (Renishaw).

The subsequent data processing workflow was provided by ImageLab software, version 3.20 (Epina GmbH Retz, Austria). Spikes (due to cosmic rays) were detected and removed using the following parameters: spike half-width − 3; threshold − 1. Next, the spectra were smoothed out using the Savitzky-Golay polynomial function, window: 7. Then, the baseline was corrected using the Eilers algorithm using the following parameters: smoothness − 10,000; asymmetry − 0.002; iterations − 7. Suspicious pixels (data without spectral noise) were masked and mainly corresponded to the oversaturated signal of the epoxide used for sample preparation.

### Data Availability

The datasets generated during and/or analysed during the current study are available in the ASEP data repository, 10.57680/asep.0587258.
